# Understanding Causal Relationships Between Imaging-Derived Phenotypes and Parkinson’s Disease: A Mendelian Randomization and Observational Study

**DOI:** 10.3390/biomedicines13030747

**Published:** 2025-03-18

**Authors:** Yichi Zhang, Min Zhong, Zhao Yang, Xiaojin Wang, Zhongxun Dong, Liche Zhou, Qianyi Yin, Bingshun Wang, Jun Liu, Yuanyuan Li, Mengyue Niu

**Affiliations:** 1 Department of Neurology and Institute of Neurology, Ruijin Hospital, Shanghai Jiao Tong University School of Medicine, Shanghai 200025, China; zyczyc@sjtu.edu.cn (Y.Z.); neuro_zhongmin@163.com (M.Z.); yangzhaoinfant@163.com (Z.Y.); zlclich-47@alumni.sjtu.edu.cn (L.Z.); 15221123532@139.com (Q.Y.); jly0520@hotmail.com (J.L.); 2 Department of Biostatistics, Clinical Research Institute, Shanghai Jiao Tong University School of Medicine, 227 South Chongqing Road, Shanghai 200025, China; wangxiaojin106@sjtu.edu.cn (X.W.); dzx5408@163.com (Z.D.); wangbingshun@sjtu.edu.cn (B.W.)

**Keywords:** Parkinson disease, neuroimaging, Mendelian randomization analysis, causality

## Abstract

**Background/Objectives:** Observational studies have suggested a correlation between brain imaging alterations and Parkinson’s disease (PD). However, data on causal relationships are still lacking. This study aimed to examine the causal relationship between brain imaging-derived phenotypes (IDPs) and PD. **Methods:** A bidirectional two-sample Mendelian randomization analysis was conducted to explore the causal association between IDPs and PD. Summary-level data for IDPs (*n* = 39,691), PD (*n* = 482,730), and PD symptoms (*n* = 4093) were obtained from genome-wide association studies of European ancestry. Clinical validation was performed in an Asian cohort, which involved healthy controls (*n* = 81), patients with idiopathic rapid-eye-movement sleep behavior disorder (iRBD) (*n* = 47), and patients with PD (*n* = 85). **Results:** We found 13 IDPs with significant causal effects on PD and seven reciprocal effects of PD on IDPs. For instance, increased median T2star in the right caudate (odds ratio = 1.23, 95% confidence interval 1.08–1.40, *p* = 0.0057) and bilateral putamen (left: odds ratio = 1.25, 95% confidence interval 1.09–1.43, *p* = 0.0056; right: odds ratio = 1.25, 95% confidence interval 1.10–1.43, *p* = 0.0056) were associated with PD. Enlargement of the left thalamus (odds ratio = 1.50, 95% confidence interval 1.14–1.96, *p* = 0.016) demonstrated causal links with PD. No reverse causal effects were detected. Observational analyses results in the Asian cohort (healthy controls, iRBD, PD) aligned with the Mendelian randomization results. **Conclusions:** Our results suggest bidirectional causal links between IDPs and PD, offering insights into disease mechanisms and potential imaging biomarkers for PD.

## 1. Introduction

Parkinson’s disease (PD) is the second most prevalent neurodegenerative disorder globally, imposing a significant health and economic burden on society. Emerging evidence suggests that PD development involves genetic, environmental, inflammatory, and hormonal factors, and so on [1,2,3,4,5,6]. However, despite extensive research, the pathogenesis of PD remains unclear, and no effective causative treatments exist. As a result of its insidious onset and prolonged clinical latency, opportunities for early neuroprotection are often missed by the time motor symptoms emerge. Therefore, identifying early diagnostic biomarkers and exploring the underlying mechanisms of PD are crucial.

Brain imaging, a non-invasive method, is essential for detecting PD at various stages by revealing changes in brain structure, connectivity, and function. Observational studies have linked brain imaging-derived phenotypes (IDPs) to PD. For example, susceptibility-weighted imaging (SWI) shows reduced high signals in the dorsal lateral substantia nigra (SN) (nigrosome 1) in patients with PD [7], aiding in the conversion from idiopathic rapid-eye-movement sleep behavior disorder (iRBD) to PD [8]. Diffusion-weighted imaging (DWI) also reveals altered connections among the SN, thalamus, and striatum in PD [9]. Additionally, research has demonstrated a close association between brain IDPs and PD subtypes and symptoms [10]. For instance, functional connectivity shows distinct patterns in tremor-dominant PD compared to posture-instability and gait-difficulty PD [11]. Regarding non-motor symptoms, hypoactivation in the right inferior frontal gyrus has been observed during response inhibition tasks, whereas hypoactivation in the left inferior frontal gyrus is linked to cognitive flexibility deficits in PD [12]. Additionally, patients with PD and RBD exhibited a trend toward a reduced mean locus coeruleus:pons ratio compared to patients with PD without RBD [13]. Depression severity in PD has also been associated with reduced gray matter volume in regions such as the thalamus and amygdala [14]. These findings highlight the importance of exploring the relationship between brain imaging and the diverse subtypes and symptoms of PD. Although these studies suggest a strong correlation between imaging findings and PD, observational studies are susceptible to confounding factors and cannot determine whether these imaging features precede or follow PD onset. Therefore, it is essential to investigate the causal role of IDPs in PD and explore their potential bidirectional relationship.

Mendelian randomization (MR) analysis, which is comparable to a naturally occurring randomized controlled trial, randomly allocates alleles during gametogenesis, making it useful for assessing causal relationships between exposure and outcome. By using single nucleotide polymorphisms as instrumental variables, MR controls for confounding factors and avoids reverse causality. In 2023, Zhu et al. used MR to study the link among three white matter lesions (i.e., white matter hyperintensities, fractional anisotropy, and mean diffusivity) and PD risk [15]. Yu et al. also recently used MR to explore the association between brain structure and PD risk [16]. However, these studies focused on structural changes and lacked data on connectivity and function. Furthermore, they did not examine causal links between IDPs and PD symptoms or severity. Therefore, a comprehensive analysis of multimodal brain imaging and its relationship with PD, motor/non-motor symptoms, and severity is needed. Understanding these causal relationships may uncover PD pathogenesis, early biomarkers, and targets for prevention.

Thus, this study aimed to examine the causal relationship between brain IDPs and PD. Specifically, the objectives were to assess the causal effects of IDPs on PD and its symptoms, and verse visa; validate the findings in an Asian cohort to determine their applicability across populations; and identify neuroimaging changes that precede PD onset, particularly in patients with iRBD. Towards this goal, we performed bidirectional two-sample MR to explore causal associations between 3370 IDPs (*n* = 39,691) and PD (*n* = 482,730), along with PD symptoms and severity (*n* = 4093) [17,18,19]. The findings were then validated in an Asian PD cohort to assess whether the results from the European patients applied to Asians. Additionally, patients with iRBD, confirmed by video-polysomnography, were included to strengthen our MR findings and explore neuroimaging changes that could serve as early biomarkers for PD.

## 2. Materials and Methods

### 2.1. Study Design

**Figure 1:**
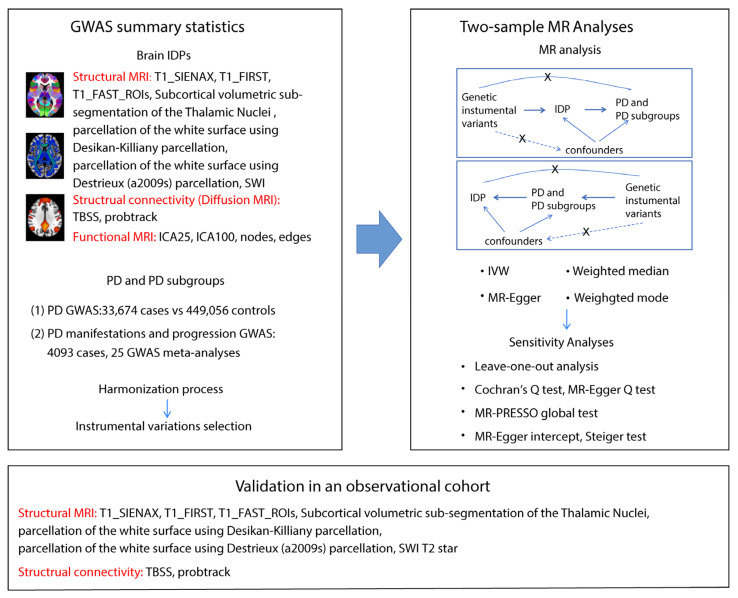
Workflow of the causal inference between IDPs and PD. The diagram shows the Mendelian randomization and observational study to explore the causal relationships between IDPs and PD. The procedure entails the selection of specific SNPs from GWAS datasets, performing MR analysis, sensitivity analyses, and observational cohort analyses. Abbreviations: T1_SIENAX: T1-weighted imaging analysis using SIENA (Structural Image Evaluation, using Normalization, of Atrophy): cross-sectional; T1_FIRST: T1-weighted imaging analysis using FMRIB’s Integrated Registration and Segmentation Tool; T1_FAST_ROI: T1-weighted imaging gray matter segmentation using FMRIB’s Automated Segmentation Tool within 139 regions of interest; SWI: susceptibility-weighted imaging; TBSS: Tract-Based Spatial Statistics style analysis; probtrack: probabilistic tractography (with crossing fiber modelling) using PROBTRACKx; ICA: independent component analysis; SNPs: single nucleotide polymorphisms; IVW: inverse-variance weighted; and MR-PRESSO: Mendelian randomization Pleiotropy RESidual Sum and Outlier.

Genome-wide association study (GWAS) summary data on IDPs and PD, including motor/non-motor symptoms and disease severity, were focused on individuals of European descent to minimize population bias. Care was taken to avoid including individuals in both exposure and outcome groups, reducing false positives. A two-sample MR analysis was then performed between exposure–outcome pairs with genetic correlation, along with a reverse MR to test for reverse causality. The primary method used was the inverse variance-weighted method, supplemented by MR-Egger, weighted median, and weighted mode methods. Sensitivity analyses were used to assess the robustness of the MR results. Finally, the MR findings were validated in our Asian cohort (healthy controls [HCs], iRBD, PD) to examine the relationship between brain IDPs and PD. In summary, GWAS data, MR analysis, and independent cohort validation were used to explore the relationship between brain IDPs and PD. The study flowchart is shown in Figure 1.

### 2.2. GWAS Datasets of Brain IDPs

Summary data were obtained from Smith et al., who conducted a genome-wide association study (GWAS) using 39,691 brain-imaged samples in the UK Biobank in 2021 [17]. To ensure reliability and reproducibility, we filtered the IDPs using the following process. First, some brain regions may be measured multiple times using different tools, leading to redundant IDPs. Therefore, we retained only the IDPs measured by the most commonly used tools. Second, we selected DWI-derived IDPs analyzed via tract-based spatial statistics (TBSS) and probabilistic tractography to cover all connectivity information. The following IDPs were included: (1) T1-weighted imaging metrics: gray matter, white matter, cerebrospinal fluid, and whole brain volumes; volumes of specific brain regions, cortical area, and thickness. (2) SWI metrics: thalamus, caudate, putamen, pallidum, hippocampus, amygdala, and accumbens. (3) DWI metrics: fractional anisotropy (FA), mean diffusivity, and intracellular volume fraction, analyzed via TBSS and probabilistic tractography. (4) Resting-state MRI: analyzed using independent component analysis. A more detailed rationale for the selection of IDPs is provided in the Supplementary Methods. A total of 3370 IDP traits were selected for the study, as outlined in Table S1 [17]. Table S2 provides detailed information on the IDP GWAS.

### 2.3. GWAS Datasets of PD and Its Manifestations and Severity

The PD GWAS data from the International Parkinson Disease Genomics Consortium 2019 release included 33,674 disease cases and 449,056 controls [18]. PD severity and symptom data were obtained from 12 longitudinal cohorts (*n* = 4093) in a GWAS conducted by Iwaki et al. in 2019 [19]. These cohorts spanned North America, Europe, and Australia and included observational studies and randomized clinical trials, which were selected based on ethical approval, informed consent, and the availability of phenotypic and genetic data from treated and untreated patients with PD. The meta-analysis incorporated 204 GWASs across 12 longitudinal cohorts. Studies were selected based on the following criteria: a minor allele frequency > 0.05, a genomic inflation factor ≤ 1.2, cohort-wide minor allele frequency variability ≤ 15%, Cochran’s Q-test *p*-value ≥ 0.05, and a sample size ≥ 1000. The final dataset included 25 studies analyzing multiple PD-related traits, including baseline binary traits such as constipation, cognitive impairment, depression, daytime sleepiness, Hoehn and Yahr (H–Y) stage 3 or worse, hyposmia, insomnia, motor fluctuation, and a Schwab and England Activities of Daily Living (SEADL) score ≤ 70. In addition, survival binary traits such as constipation, cognitive impairment, depression, daytime sleepiness, H–Y stage 3 or worse, hyposmia, and insomnia were assessed. Continuous traits included H–Y staging, the Unified Parkinson’s Disease Rating Scale (UPDRS) total score and scores for its four parts, the Mini-Mental State Examination (MMSE) score, the Montreal Cognitive Assessment (MoCA) score, and SEADL scores. This ensured a robust dataset for disease progression assessment. Importantly, we carefully selected non-overlapping datasets from populations with the same ancestors. There was no overlap between IDP and PD GWAS data because of their diverse origins. Details are provided in Table S2.

### 2.4. Selection of Instrument Variants and Harmonization of Single Nucleotide Polymorphisms

In this two-sample MR study, instrumental variables (IVs) were selected based on three core criteria: (1) strong association with exposure (*p* < 5 × 10^−8^); (2) independence from confounders, verified using Phenoscanner V2; and (3) pathway-specific influence on outcome (*p* > 5 × 10^−5^). Detailed criteria are listed in the Supplemental Methods. SNP independence was determined using PLINK clumping with the 1000 Genomes Project as a reference for linkage disequilibrium trimming (R^2^ < 0.001 and LD > 10,000 kb). Further quality control included calculating the F-statistic for each IV, excluding those with F < 10. After harmonization and removal of palindromic and ambiguous SNPs, the final IVs were used in MR analyses. These IVs are listed in Tables S3 and S4.

### 2.5. Bidirectional Two-Sample MR Analyses

The MR analysis consisted of two parts: forward MR assessed whether IDPs causally influenced PD, severity, and symptoms, whereas reverse MR examined whether reverse causal relationships existed in the positive forward MR results. Using the study by Burgess et al. as guide, inverse variance-weighted MR was applied as the primary method. We began with a fixed-effects model and calculated Cochrane’s Q statistic using a chi-square test. If *p* < 0.05 (indicating heterogeneity), a multiplicative random-effects model was used. To ensure reliability, additional analyses were conducted using MR-Egger, weighted median, and weighted mode methods. The Wald Ratio was applied when only one genetic instrument was available.

### 2.6. Sensitivity Analyses

Sensitivity analyses were performed to verify the results of MR study with four methods: Cochran’s Q test, leave-one-out analysis, MR-Egger intercept, and MR-PRESSO. Cochrane’s Q test was employed for the heterogeneity investigation, and the MR-Egger intercept and MR-PRESSO global test were used for the pleiotropy assessment. A genetic variant was referred to as pleiotropic if it had associations with more than one risk factor on different causal pathways. The Steiger test was subsequently conducted to prevent potential reverse causation. The leave-one-out test was performed to determine if a single SNP could determine the MR result. After these analyses, sub-optimal single nucleotide polymorphisms were excluded, and another MR analysis was performed to obtain the final results.

### 2.7. Observational Study Participants

A total of 213 participants, including 81 HCs, 47 patients with iRBD, and 85 patients with PD, were recruited from the Department of Neurology, Ruijin Hospital, Shanghai, China. Participants met the following criteria: (1) age 45–80 years, (2) absence of dementia or psychiatric disorders, (3) no history of intracranial surgery or traumatic brain injury, (4) no familial history of PD or other neurological disorders, and (5) no systemic inflammatory or severe primary diseases. PD diagnoses were made by at least two neurologists based on current Movement Disorders Society criteria. IRBD diagnoses were confirmed by video-polysomnography following the International Classification of Sleep Disorders-II criteria. All patients with iRBD were examined to exclude motor signs of parkinsonism or secondary causes.

### 2.8. Brain Magnetic Resonance Imaging Acquisition and Processing

Structural and functional images were acquired using 3T Siemens scanners (Siemens Healthineers, Erlangen, Germany) with a 12-channel head coil at the Department of Radiology, Ruijin Hospital. T1-weighted images were obtained using a 3D magnetization-prepared rapid acquisition gradient-echo sequence, and structural connectivity was assessed via diffusion-weighted imaging scanning. Detailed settings are provided in the Supplementary Methods. Imaging was processed using the UKB pipeline (https://git.fmrib.ox.ac.uk/falmagro/UK_biobank_pipeline_v_1, accessed on 4 October 2023). Positive IDPs in the MR study were analyzed in the observational cohort. Specifically, given the potential for extreme values in the T2star values in a brain region, medians and interquartile ranges (IQRs) were used as they are less sensitive to outliers. Regarding data at the participant level, when the normality assumption and homogeneity of variances were met, we reported means and standard errors. Otherwise, we presented medians and interquartile ranges.

### 2.9. Statistical Analysis

All MR analyses were performed using TwoSampleMR (version 0.5.7) and MRPRESSO in R Software 4.3.1. (https://www.R-project.org). All statistical analyses were performed with car (version 3), readxl (version 1.4.3), nlme, multcomp, pROC, tidyverse, dunn.test, PMCMR, FSA, DescTools, and dplyr packages in R. An exposure–outcome association was considered positive if (1) *p* < 0.05 in the inverse variance-weighted method and (2) the direction of results from MR-Egger, weighted median, and weighted mode were consistent with inverse variance-weighted. The Bonferroni method was used for multiple comparison corrections of instrumental variations, and the Benjamini–Hochberg false discovery rate (FDR) method was used for multiple comparison corrections of different IDPs. In the observational study, clinicodemographic characteristics were analyzed using chi-square tests (sex) or analysis of variances (age). Normality was tested using Q-Q plot, while homoscedasticity was tested using Levene’s test (Figure S1 and Table S13). A linear mixed-effects model was used to analyze group differences in T2star values or the volume of specific brain regions, accounting for repeated measures across different brain regions within each subject. A Tukey–Kramer adjustment was applied to these multiple comparisons. Detailed analyses are shown in the Supplementary Methods. All hypothesis tests were two-tailed, and a significance level of α = 0.05 was used throughout. Distribution of standardized residuals were tested Q-Q plots. Tests on residuals were shown in Figure S2. Receiver operating characteristic curve (ROC) analysis was used to evaluate discriminative power.

## 3. Results

### 3.1. Forward MR: The Putative Causal Effects of IDPs on PD

**Figure 2:**
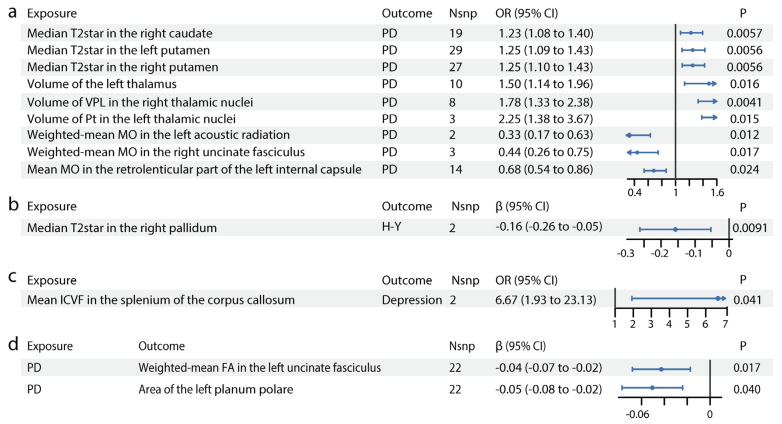
Causal relationships between IDPs and PD. Forest plots show the significant causal effects of IDPs on PD (**a**), PD severity (**b**), and PD manifestations (**c**) with *p* < 0.05. The forest plots demonstrate significant causal effects of alternations in median T2star in the striatum and volumes of the thalamus, as well as its subregions VPL and Pt on PD (**a**). Furthermore, the data suggest that altered median T2star in the pallidum might be causally associated with PD severity (**b**), while the mean ICVF in the splenium of the corpus callosum may be causally associated with PD depression (**c**). (**d**) Forest plots show significant causal effects of PD on IDPs. Data suggest PD might have causal effects on weighted-mean FA in the tract UF and area of the left planum polare. Each circle in the graph represents an inverse-variance weighted estimate. The horizontal line represents the 95% confidence intervals (CIs) for the estimates. For binomial outcomes, MR estimates are reported as odds ratios (ORs) along with their corresponding 95% Cls. For continuous outcomes, the MR estimates are reported as betas with their 95% CIs. Abbreviations: nsnp: number of SNPs; P: *p* value after Bonferroni and false discovery rate correction; H-Y: Hoehn–Yahr stage; VPL: ventral posterolateral nucleus; Pt: paratenial nucleus; MO: diffusion tensor mode; ICVF: intracellular volume fraction; FA: fractional anisotropy.

As shown in Figure 1, IDP GWAS data were extracted from the UK Biobank imaging cohort (*n* = 39,691), PD GWAS from the International Parkinson’s Disease Genomics Consortium (*n* = 482,730) and PD manifestation GWAS (*n* = 4093). We identified 13 IDPs with significant causal effects on PD (Figure 2, Table S5). The scatter plots are shown in Figure S3. In susceptibility-weighted imaging analysis, increased median T2star in the right caudate was associated with higher PD risk (odds ratio [OR] = 1.23, 95% CI = 1.08–1.40, *p* = 0.0057) (Figure 2a). An elevated T2star was also observed bilaterally in the putamen (left: OR = 1.25, 95% CI = 1.09–1.43, *p* = 0.0056; right: OR = 1.25, 95% CI = 1.10–1.43, *p* = 0.0056). In T1-weighted imaging, increased left thalamus volume (OR = 1.50, 95% CI = 1.14–1.96, *p* = 0.016) contributed to PD incidence (Figure 2a). Given the independent function of the thalamic subregions, we further analyzed the volumetric segmentation of thalamic nuclei involved in PD. Enlarged paratenial nucleus (Pt; OR = 2.25, 95% CI = 1.38–3.67, *p* = 0.015) and ventral posterolateral nucleus (VPL; OR = 1.78, 95% CI = 1.33–2.38, *p* = 0.0041) showed direct causal links with PD (Figure 2a). In diffusion-weighted imaging analysis, increased mode of anisotropy in the retrolenticular part of the left internal capsule reduced PD risk (OR = 0.68, 95% CI = 0.54–0.86, *p* = 0.024). Higher median mode of anisotropy in the left acoustic radiation (OR = 0.33, 95% CI = 0.17–0.63, *p* = 0.012) and the right uncinate fasciculus (UF) (OR = 0.44, 95% CI = 0.26–0.75, *p* = 0.017) were also causally associated with reduced PD risk.

### 3.2. Forward MR: The Putative Causal Effects of IDPs on PD Severity and Symptoms

For PD severity, increased median T2star in the right pallidum appeared to reduce disease severity, as indicated by its association with a lower H–Y Scale score (β = −0.16, 95% CI = −0.26–−0.05, *p* = 0.0091, Figure 2b). The causal links between IDPs and PD symptoms, including motor symptoms (dyskinesias, motor flux, UPDRS scores) and non-motor symptoms (constipation, dementia, depression, hyposmia, sleep disorders), were also analyzed. In diffusion-weighted imaging analysis, higher intracellular volume fraction in the splenium of the corpus callosum was linked to an increased risk of depression in patients with PD (OR = 6.67, 95% CI = 1.93–23.13, *p* = 0.041, Figure 2c). No significant results were found for other PD symptoms. Details are provided in Table S5.

### 3.3. Forward MR: The Putative Causal Effects of PD and Its Severity and Symptoms on IDPs

We identified significant causal relationships between seven other IDPs and PD (Figure 2d, Table S6). The scatter plots are shown in Figure S3. The mean FA in the left UF (β = −0.04, 95% CI = −0.07–−0.02, *p* = 0.017) was mildly reduced in PD. The area of the planum polare (β = −0.05, 95% CI = −0.08–−0.02, *p* = 0.040, Figure 2d), located anterior to the transverse temporal gyrus, was also slightly decreased in PD.

### 3.4. Reverse MR

As shown in Tables S5 and S6, IDPs showed no significant reverse causal relationships with PD and its severity or symptoms detected in the forward MR study.

### 3.5. Sensitivity Analyses

Sensitivity analyses confirmed the robustness of the MR results. The leave-one-out test indicated that no single SNP significantly influenced the causal estimates (Figure S4). The Steiger test found no evidence of reverse causation (Tables S7 and S8). Heterogeneity was assessed using the Cochrane’s Q tests, while pleiotropy was evaluated with the MR-Egger intercept and MR-PRESSO global tests. All tests showed no significant heterogeneity or pleiotropy (Tables S9–S12).

### 3.6. Concordance Between Observational Study Results and MR Analysis Results

**Figure 3:**
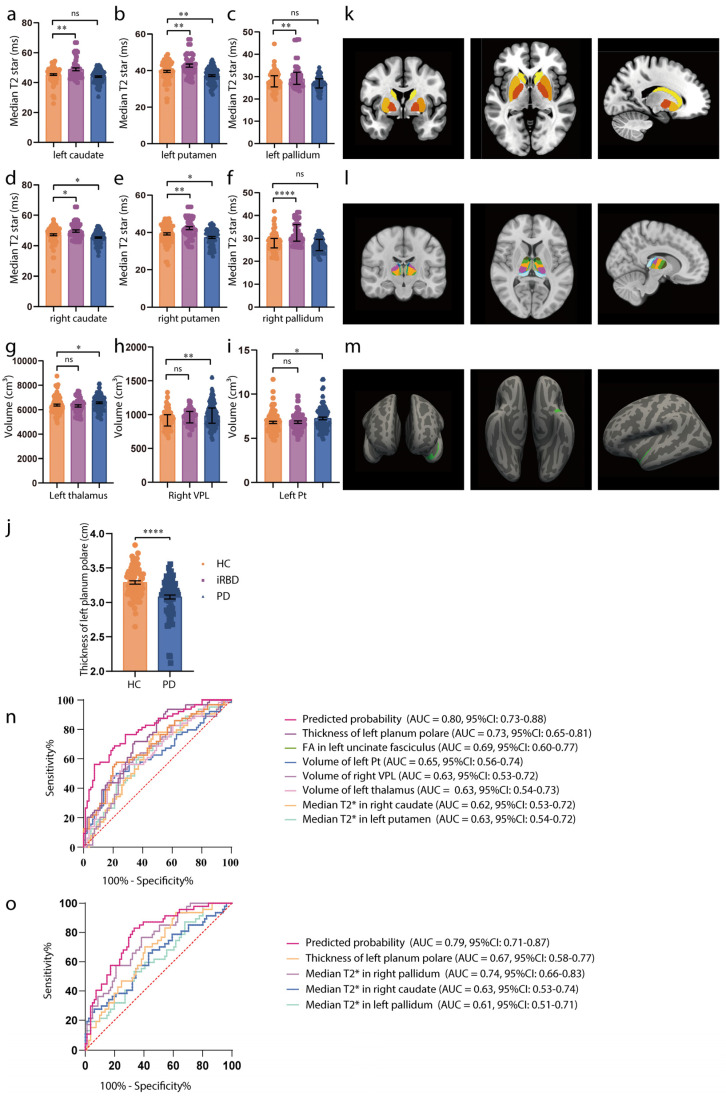
Associations of brain IDPs and PD in the observational study. (**a**–**f**) Differences in median T2star in the caudate, putamen, and pallidum across HCs (orange), patients with iRBD (purple), and patients with PD (blue). Patients with iRBD have higher T2star values, while those with PD show lower values for both caudate sides. Similar results are observed in both sides of the pallidum and putamen. (**g**–**i**) Volumes of the thalamus, Pt, and VPL in HCs (orange), participants with iRBD (purple), and participants with PD (blue). The thalamus volume is larger in both the iRBD and PD groups, with consistent results in the left Pt and right VPL. (**j**) Thickness of the left planum polare in HCs (orange), and participants with PD (blue). The thickness of the superior planum polare is reduced in patients with PD. (**k**–**m**) MRI pattern diagrams corresponding to (**a**–**j**). For normally distributed variables with homogenous variances, mean and SEM are used for precision and dispersion measures, setting error bars as the SEM. For non-normally distributed variables with heterogenous variances, median and interquartile ranges (IQRs) are used for precision and dispersion measures, setting the error bars as the IQRs. We used a linear mixed-effects model. A Tukey–Kramer adjustment was applied to these multiple comparisons. The discriminative power of the values is evaluated using ROC curve analysis. (**n**) ROC curve distinguishing HCs from early-stage PD participants. (**o**) ROC curve distinguishing HCs from iRBD participants. Significance codes: “ns” for no significance; * *p* < 0.05; ** *p* < 0.01; **** *p* < 0.0001. Abbreviations: FA, fractional anisotropy; HCs, healthy controls; iRBD, idiopathic rapid-eye-movement sleep behavioral disorder; MRI, magnetic resonance imaging; PD, Parkinson’s disease; ROC, receiver operating characteristic; and VPL, ventral posterolateral nucleus.

Table 1 presents the clinicodemographic characteristics of the PD (*n* = 85), iRBD (*n* = 47), and HC (*n* = 81) participants. iRBD is recognized as a prodromal phase of synucleinopathies, with a high conversion rate to PD. The MR study revealed that increased striatum T2star values and an enlarged thalamus, particularly subregions Pt and VPL, were causally linked with PD. We compared T2star values and thalamus volume in HCs, patients with iRBD, and patients with PD. As shown in Figure 3a–f,k, participants with iRBD had higher median T2star values, while those with PD showed a decrease in values of both caudate sides (HC vs. iRBD: left caudate: t = 3.65, *p* = 0.0010, right caudate: t = 2.73, *p* = 0.0185; HC vs. PD: left caudate: t = −1.65, *p* = 0.2288, right caudate: t = −2.41, *p* = 0.0437). Similar results were observed in both sides of the pallidum and putamen (HC vs. iRBD: left pallidum: t = 3.46, *p* = 0.0019, right pallidum: t = 5.52, *p* < 0.0001; HC vs. PD: left pallidum: t = −1.50, *p* = 0.2954, right pallidum: t = −1.53, *p* = 0.2782; HC vs. iRBD: left putamen: t = 3.35, *p* = 0.0028, right putamen: t = 3.33, *p* = 0.0030; HC vs. PD: left putamen: t = −2.97, *p* = 0.0092, right putamen: t = −2.41, *p* = 0.0442). Thalamus volume was larger in both the iRBD and PD groups (HC vs. iRBD: t = 0.186, *p* = 0.9760; HC vs. PD: t = 2.305, *p* = 0.0421), with consistent results in the left Pt and right VPL (left Pt: HC vs. iRBD: t = 0.14, *p* = 0.9892, HC vs. PD: t = 2.4, *p* = 0.0453; right VPL: HC vs. iRBD: t = 1.24, *p* = 0.432, HC vs. PD: t = 2.97, *p* = 0.0093) (Figure 3g–i,l). Additionally, the observational study showed reduced thickness of the superior planum polare (t = −5.51, *p* < 0.0001, Figure 3j,m) in patients with PD, which is consistent with the MR results. Detailed values of statistical tests and *p*-values were shown in Table S13.

ROC analysis was used to evaluate whether these imaging markers could serve as early PD diagnostic biomarkers (H–Y score ≤ 2). The results demonstrated moderate diagnostic accuracy for individual biomarkers (AUC = 0.62–0.73, *p* < 0.05) and improved accuracy when combining biomarkers (AUC = 0.80, 95% CI = 0.73–0.88, *p* < 0.0001; Figure 3n). For distinguishing HCs from iRBD individuals, the combined biomarkers showed an improved AUC of 0.79 (95% CI = 0.71−0.87, *p* < 0.0001; Figure 3o).

## 4. Discussion

The current study identified 13 IDPs with significant evidence of potential causal effects on PD, as well as potential causal effects of PD on seven IDPs. Additionally, validation in an Asian cohort (HCs, iRBD, and PD) yielded consistent findings, offering valuable insights into PD pathogenesis and early diagnostic strategies and paving the way for advancements in disease management and prognosis. To our best knowledge, this is the first MR analysis investigating causal connections between multi-modal imaging (brain structure, connectivity, and function) and PD, including PD motor/non-motor symptoms and disease severity.

In the MR analysis, we identified susceptibility-weighted imaging features associated with increased PD risk. Susceptibility-weighted imaging measures brain tissue magnetic susceptibility, primarily influenced by iron. In the UK Biobank IDP GWAS, median T2star values were analyzed in 14 major subcortical grey matter structures, where elevated T2star levels indicated reduced iron deposition. The results demonstrated that higher T2star levels in the caudate and putamen were causally linked to increased PD risk, whereas lower T2star values correlated with higher H–Y scores. This suggests that reduced iron deposition in these regions may increase PD susceptibility, whereas higher iron accumulation could exacerbate PD severity. This supports the growing interest in iron deposition as a key factor in PD pathogenesis [20]. Extensive evidence indicates that during the progression of PD, iron is specifically alternated in various brain regions, notably the substantia nigra (SN). This disruption contributes to neurodegenerative processes, including ferritinophagy and lipid peroxidation, ultimately triggering ferroptosis (an iron-dependent form of cell death implicated in PD pathology) [21,22,23]. Given the distinct patterns of iron accumulation across brain regions, neuroimaging techniques have shown promise in detecting early-stage PD by capturing these changes. The current findings were consistent with another MR study that reported increased T2star as potentially causally associated with heightened PD risk [24]. However, that study interpreted these findings as evidence of increased iron levels contributing to PD pathogenesis. This interpretation may be misleading, as higher T2star values indicate lower iron content. Moreover, this study focused solely on SWI, without considering causal links between multimodal IDPs and PD symptoms or severity, which are crucial for understanding disease progression. Several observational studies have reported higher iron deposition in the SN [25,26]. As for the striatum, previous studies have reported inconsistent findings regarding iron accumulation in the striatum of PD patients. Some observational studies have shown increased iron deposition in the striatum [27,28,29], whereas others have reported no significant changes or even reduced iron content [30,31]. These discrepancies may stem from methodological differences, including variations in imaging modalities (e.g., SWI vs. quantitative susceptibility mapping [QSM]), PD subtypes, and disease stages analyzed across studies.

To better interpret these conflicting findings, we analyzed T2star values in our observational cohort of healthy controls, iRBD participants, and PD patients, as iRBD is considered a prodromal phase of PD. Our results revealed increased T2star values in the striatum during iRBD, suggesting reduced iron levels, followed by a decrease in T2star values in PD indicating iron accumulation. This suggests a dynamic redistribution of iron across different PD stages. In the classic model of PD circuitry, dopaminergic projections originate from the SN and extend to the caudate, putamen, and pallidum [32]. Prior cellular studies demonstrated that, during early dopaminergic neuron degeneration, iron is initially redistributed, leading to increased iron in the SN, whereas axonal projections to the striatum show transient reductions. Lashuel et al. carried out the quantitative mapping of iron distribution in subcellular compartments using synchrotron X-ray fluorescence nano-imaging and Particle-Induced X-ray Emission, finding a decreased iron levels in neurites and distal ends of neurons, whereas they were increased in cell bodies in PD models on PC12 dells [33]. Consistently, Ortega et al. overexpressed α-synuclein (a hallmark of PD) in PC12 cells and primary rat midbrain neurons and observed iron redistribution with accumulation in the perinuclear region [34]. We propose that an initial redistribution of iron occurs during the early degeneration of dopaminergic neurons, leading to increased iron in the perinuclear region of the SN (cell body) and decreased iron in the axons of the caudate and putamen (projections). As the disease progresses, iron overload spreads across all cellular compartments, leading to increased iron levels in both the SN and striatum. Future studies are needed to verify iron redistribution during different PD stages and explore the underlying mechanisms.

Regarding T1 imaging analyses in the MR study, the current study observed that an enlarged left thalamus volume was a potential causal risk factor for PD. The observational study showed progressive thalamic enlargement from HCs to patients with iRBD and PD. However, findings regarding whole thalamus morphology have varied. One study reported thalamic atrophy in PD [35], while others found no significant differences [36,37]. More recent studies have confirmed thalamic enlargement in PD [38,39,40], with evidence of progressive volume increase as the disease advances [40]. Additionally, increased thickness and more expansive surface areas have been observed in PD, which is consistent with increased volumes [38,39]. These inconsistencies may be attributed to differences in study design and methodology. Earlier studies often used lower-resolution MRI or assessed whole-thalamus volume, potentially masking out region-specific changes. Given the heterogeneity in thalamic subnuclei function, we leveraged high-resolution imaging and further assessed structural thalamic morphology at the subnuclei level. In the MR study, Pt and VPL emerged as two predominant subregions. This was corroborated by findings in the observational cohort analysis, showing progressive volume increases from HCs to iRBD and finally to PD. Our findings are consistent with previous observational findings of enlargement of these subregions [38,39]. However, the causative mechanisms remain unclear. We propose that compensatory mechanisms might explain these results. As a key node in the cortico–striato–thalamo–cortical circuit, the thalamus plays a crucial role in transferring motor, associative, and limbic signals between the cortex and subcortical nuclei [32]. Thus, thalamic enlargement could reflect adaptive compensatory responses, possibly to maintain or enhance neural function in response to underlying pathology. Furthermore, the thalamus participates in the cerebello–thalamo–cortical loop, which may compensate for degeneration in the nigrostriatal system [41]. Early thalamic hypertrophy may result from hyperactivity in the cerebellothalamic circuit, particularly linked to Parkinson’s tremor. Future studies incorporating longitudinal follow-ups and multimodal imaging are necessary to further investigate the role of thalamic volume changes in PD progression.

Another interesting finding in our MR study is that elevated mean intracellular volume fraction in the splenium of corpus callosum (CC) might have a causal relationship with PD-related depression. The CC is the most prominent white matter pathway providing information transfer between the two hemispheres. The splenium connects association areas of the parietal and temporal lobes (anterior splenium) and occipital lobes (posterior splenium). Observational studies have illustrated a greater FA in the splenium of the CC in patients with major depression disorder than in that of healthy controls, and they further identified a positive correlation between FA values in the splenium of the CC and anxiety, which is one of the three core symptoms (anhedonia, anxiety, and psychomotor retardation) involved in depression [42].

In the MR analysis to determine whether PD was a causal factor for IDPs, PD was found to possibly contribute to a lower mean FA in the left UF and decreased surface area of the left planum polare. The UF is a key tract connecting the inferior frontal gyrus and the anterior temporal lobe regions. Several researchers observed widespread demyelination and degeneration of the UF in patients with PD [43,44,45,46]. These structural changes in the UF could potentially account for the presence of depressive symptoms and anxiety commonly observed in individuals with PD. Our findings highlighted the significant influence of PD on sensory alterations, with a reduced surface area in the left planum polare of the superior temporal gyrus, associated with the primary auditory cortex [47,48]. These findings shed light on the potential underlying mechanisms linking PD pathology to both emotional disturbances and sensorimotor alterations.

This study presents compelling evidence that specific IDPs have causal relationships with PD and may serve as diagnostic or predictive biomarkers of PD risk, severity, and symptoms. First, imaging biomarkers may aid in early PD risk identification. The ROC analysis suggests that combined IDP markers improve diagnostic accuracy, with an AUC of up to 0.80 for early PD detection. Using a composite biomarker approach may be particularly useful in distinguishing HCs from patients with PD or high-risk groups, such as patients with iRBD. Second, imaging biomarkers may serve as prognostic indicators of PD progression and severity. The study found that higher median T2star values in the right pallidum were associated with lower disease severity, as indicated by a reduced H–Y score. Third, imaging biomarkers help predict and manage PD-related symptoms. For instance, higher intracellular volume fraction in the splenium of the corpus callosum was causally linked to an increased risk of depression in PD. This suggests that imaging biomarkers can guide interventions for patients at higher risk for PD-associated depression.

The strengths of the present study are substantial. First, the two-sample MR design minimized the potential for confounding factors and reverse causation, allowing us to explore underlying mechanisms and identify causal relationships between IDPs and PD. The large GWAS sample size reduced bias and increased statistical power. Second, the inclusion of comprehensive multi-modal neuroimaging data, along with detailed PD symptoms and severity, added depth to our findings. Third, validation in an Asian cohort broadened the generalizability of our results. We included patients with video-polysomnography-confirmed iRBD to better understand IDP changes in the prodromal stage of PD. Additionally, this study provided new insights into potential imaging biomarkers for predicting and diagnosing early PD. However, our study also had limitations. First, Winner’s curse bias can occur when the same GWAS data are used to select instrumental variables and estimate their association with the exposure. Avoiding Winner’s curse was balanced with minimizing precision loss due to smaller sample sizes [49]. Moreover, although we performed sensitivity analyses, pleiotropy and heterogeneity could not be avoided in the MR study. Second, selection bias was inevitable in both the UK Biobank Imaging cohort and PD cohorts. Further, the relatively small sample size of the observational cohort might have decreased the statistical power. Longitudinal studies are needed to validate our observations and explore the mechanisms underlying these relationships.

## 5. Conclusions

In conclusion, our study provides strong evidence for the bidirectional causal relationships between brain IDPs and PD. We identified several IDPs, including T2star values in the caudate and putamen that are causally associated with PD risk and severity. Thalamic volume changes were linked to disease progression, suggesting adaptive compensatory mechanisms in response to neurodegeneration. This is the first study to apply MR to assess the causal role of brain IDPs across different PD symptoms, including non-motor symptoms and disease severity. Regarding the limitation of the relatively small sample size in the observational cohort and symptom GWAS, future research should focus on validating these findings in larger, more diverse longitudinal cohorts and further investigating the role of IDPs in PD-related symptoms. These findings contribute to a deeper understanding of PD pathogenesis and may serve as the foundation for developing early biomarkers for PD diagnosis. Clinically, these biomarkers could facilitate more accurate diagnostics and enable tailored treatment strategies, particularly in detecting early-stage PD before motor symptoms appear.

## Figures and Tables

**Table 1:** Clinicodemographic characteristics of the participants in the observational study.

	HC (*n* = 81)	iRBD (*n* = 47)	PD (*n* = 85)	Test Statistic	*p*-Value
Age (year)	62.77 ± 8.10	67.1 ± 5.68	67.87 ± 6.97	F = 11.53	<0.0001
Sex, *n*				χ² = 0.1348	0.94
Male	40 (49%)	23 (49%)	44 (52%)		
Female	41 (51%)	24 (51%)	41 (48%)		
Disease duration (m)	-	50.65 ± 15.74	97.49 ± 36.24		
H–Y stage	-	-	1.97 ± 0.80		
UPDRS I score	-	-	9.02 ± 5.46		
UPDRS II score	-	-	11.84 ± 6.33		
UPDRS III score	-	-	33.13 ± 15.19		

The clinicodemographic characteristics of the participants are analyzed using the chi-square test or ANOVA, depending on the type of dependent variables. Data are presented as the mean ± SD for continuous variables and as frequency (%) for categorical variables. Abbreviations: HC, healthy control; H–Y, Hoehn and Yahr stage; iRBD, idiopathic rapid-eye-movement sleep behavior disorder; PD, Parkinson’s disease; UPDRS, MDS Unified Parkinson Disease Rating Scale.

## Data Availability

Genome-wide summary statistics for brain imaging phenotypes used in the current study are available at https://open.win.ox.ac.uk/ukbiobank/big40/ (accessed on 4 October 2023) [17]. Genome-wide summary statistics for Parkinson’s disease used in the current study are available at IEU OpenGWAS project https://gwas.mrcieu.ac.uk/datasets/ieu-b-7/ (accessed on 4 October 2023) [18]. Genome-wide summary statistics for Parkinson’s disease manifestations used in the current study are available at https://pdgenetics.shinyapps.io/pdprogmetagwasbrowser/ (accessed on 4 October 2023) [19].

## Supplementary Materials

The following supporting information can be downloaded at: https://www.mdpi.com/article/10.3390/biomedicines13030747/s1, Figure S1. Quantile-Quantile (QQ) plots for normality assessment. The plots compare the quantiles of the observed data (y-axis) against the expected quantiles from a normal distribution (x-axis). A linear pattern along the diagonal line indicates that the data follows a normal distribution, while deviations suggest departures from normality; Figure S2. Quantile-Quantile (QQ) plots for assessing the normality of residuals. The plots compare the quantiles of the model residuals (y-axis) against the expected quantiles from a normal distribution (x-axis). If the residuals follow a normal distribution, the points should align closely along the diagonal reference line. Deviations from this line indicate potential departures from normality, which may suggest violations of the normality assumption in the model; Figure S3. Scatter plots of the forward Mendelian randomization study on the causal associations between IDPs and PD. Scatter plots show the effects of single nucleotide polymorphisms (SNPs) on IDPs against their effects on PD. The model-fit lines indicate the MR estimates of different MR methods; Figure S4. Leave-one-out plots of the forward Mendelian randomization study on the causal associations between IDPs and PD. Leave-one-out sensitivity analyses were used to identify potentially influential single nucleotide polymorphisms that exerted significant impacts on the associations. The horizontal line represents the 95% confidence intervals (CI) for the estimates; Table S1: Names and descriptions of the IDPs in this study; Table S2: Information for the GWAS summary data used in this study; Table S3: IVs included in MR analysis regarding the putative causal effects of IDP on PD and its symptoms; Table S4: IVs included in MR analysis regarding the putative causal effects of PD and its symptoms on IDPs; Table S5: MR analysis results regarding the putative causal effects of IDP on PD and its symptoms; Table S6: MR analysis results regarding the putative causal effects of PD and its symptoms on IDPs; Table S7: Steiger test for significant results of the MR analysis regarding the putative causal effects of IDP on PD and its symptoms; Table S8: Steiger test for significant results of the MR analysis regarding the putative causal effects of PD and its symptoms on IDPs; Table S9: Heterogeneity test for significant results of the MR analysis regarding the putative causal effects of IDP on PD and its symptoms; Table S10: Heterogeneity test for significant results of the MR analysis regarding the putative causal effects of PD and its symptoms on IDPs; Table S11: Pleiotropy test for significant results of the MR analysis regarding the putative causal effects of IDP on PD and its symptoms; Table S12: Pleiotropy test for significant results of the MR analysis regarding the putative causal effects of PD and its symptoms on IDPs; Table S13: Results of the observational study; and Supplementary Methods.

## Abbreviations

The following abbreviations are used in this manuscript:

ANOVAs	Analysis of variances
CC	Corpus callosum
FA	Fractional anisotropy
FDR	False discovery rate
GWAS	Genome-wide association studies
HCs	Healthy controls
IDPs	Imaging-derived phenotypes
iRBD	Idiopathic rapid-eye-movement sleep behavior disorder
IVs	Instrumental variables
MR	Mendelian randomization
OR	Odds ratio
PD	Parkinson’s disease
ROC	Receiver operating characteristic curve
UPDRS	Unified Parkinson Disease Rating Scale
SN	Substantia nigra
UF	Uncinate fasciculus
VPL	Ventral posterolateral nucleus
