# Supplementary material for: Understanding Causal Relationships Between Imaging-Derived Phenotypes and Parkinson’s Disease: A Mendelian Randomization and Observational Study
Source: Biomedicines. 2025 Mar 18;13(3):747. doi: 10.3390/biomedicines13030747 (PMC11940266; doi:10.3390/biomedicines13030747)
Supplement: Supplementary file 1 [file biomedicines-13-00747-s001.zip › Supplementary Figures.pdf]

Supplementary Figures

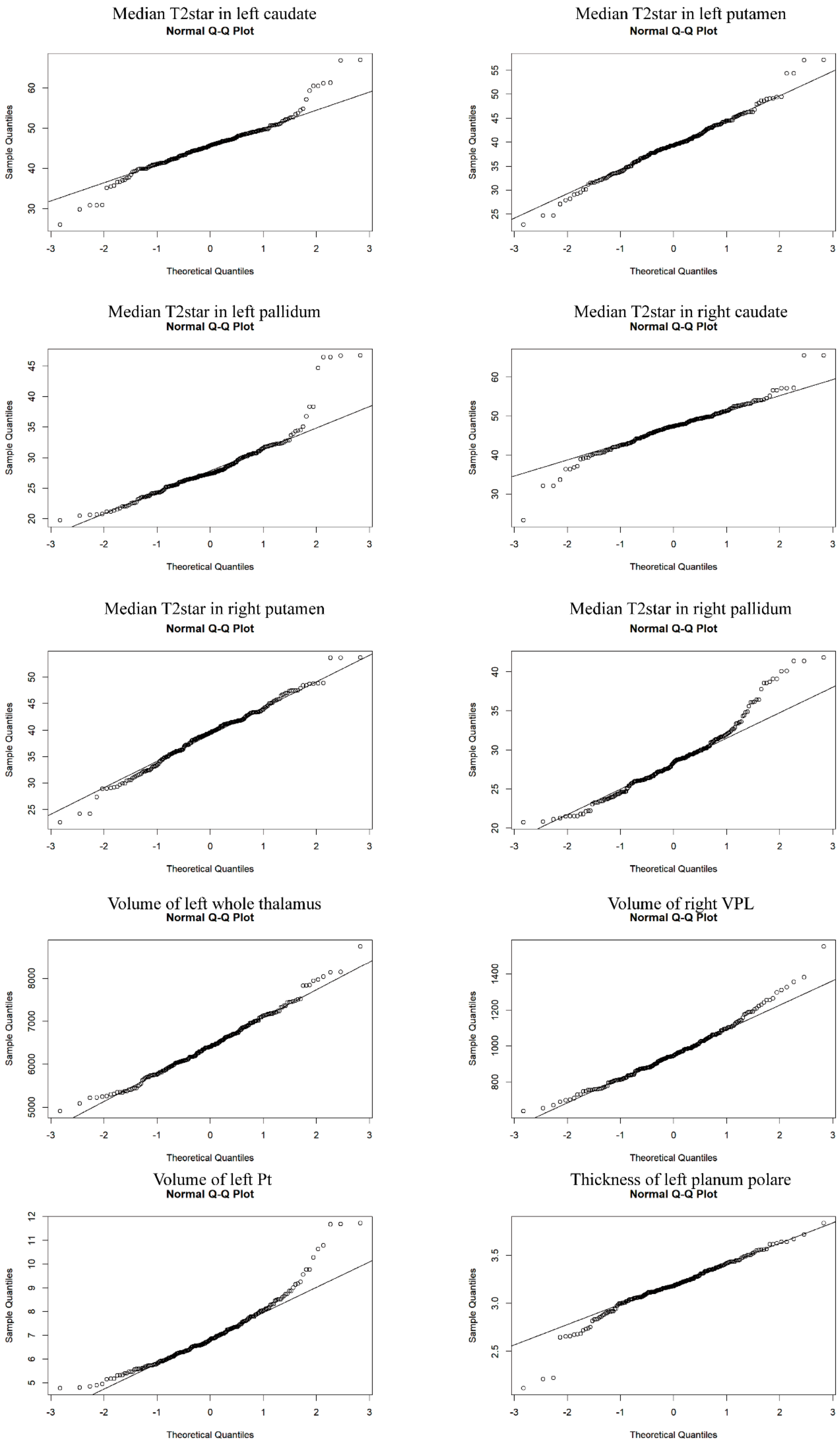

Figure S1. Quantile-Quantile (QQ) plots for normality assessment. The plots compare the quantiles of the observed data (y-axis) against the expected quantiles from a normal distribution (x-axis). A linear pattern along the diagonal line indicates that the data follows a normal distribution, while deviations suggest departures from normality.

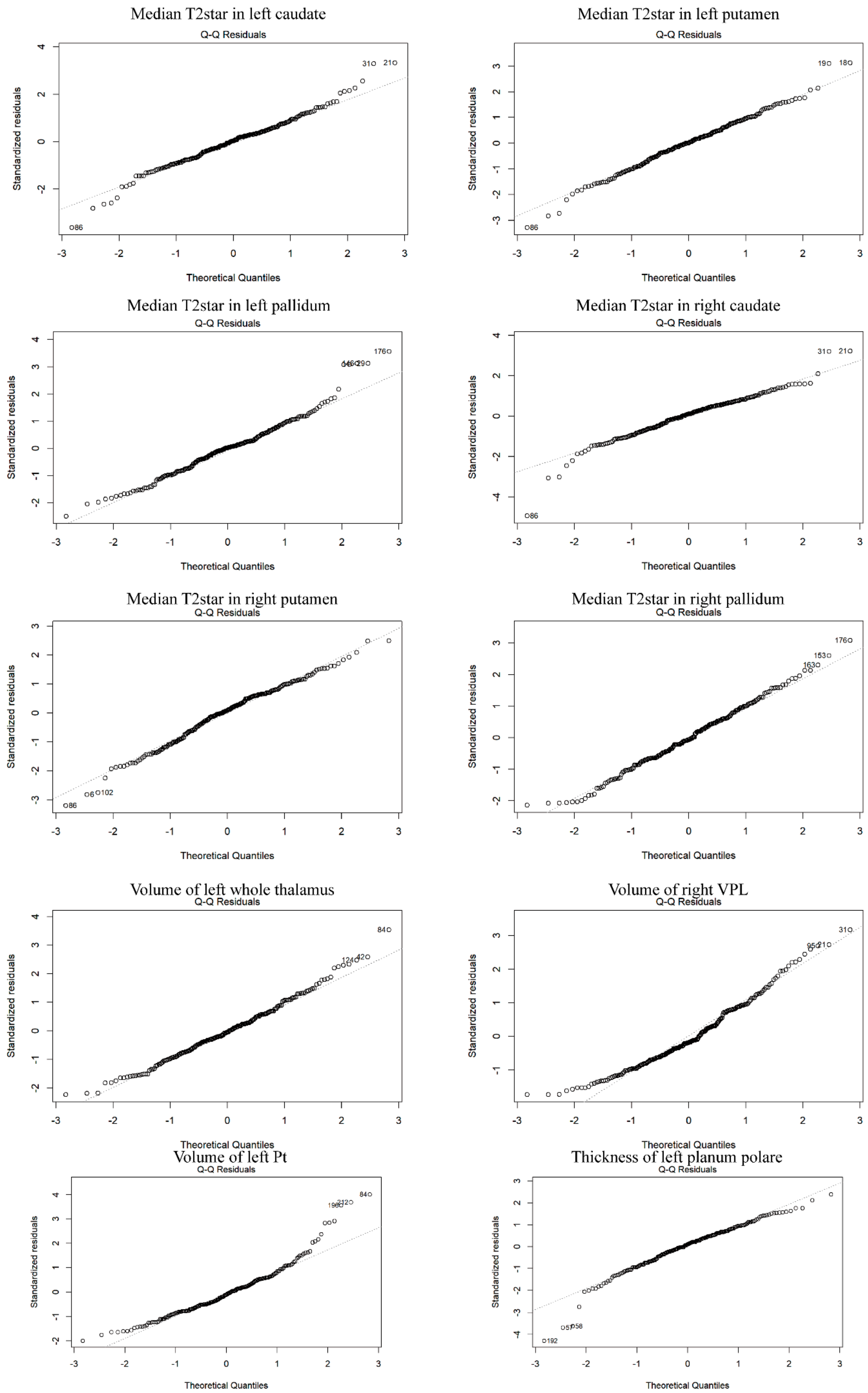

Figure S2. Quantile-Quantile (QQ) plots for assessing the normality of residuals. The plots compare the quantiles of the model residuals (y-axis) against the expected quantiles from a normal distribution (x-axis). If the residuals follow a normal distribution, the points should align closely along the diagonal reference line. Deviations from this line indicate potential departures from normality, which may suggest violations of the normality assumption in the model.

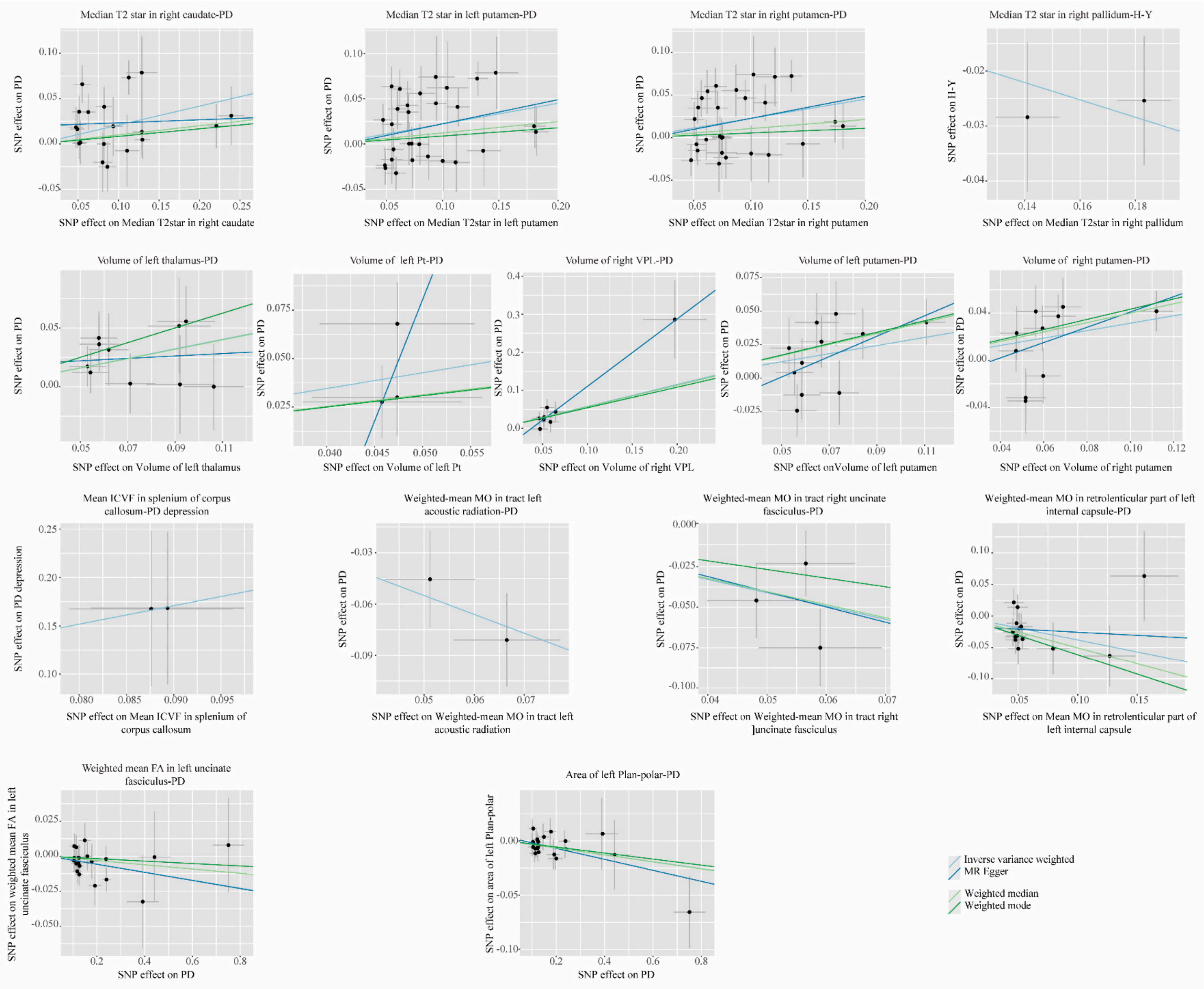

Figure S3. Scatter plots of the forward Mendelian randomization study on the causal associations between IDPs and PD. Scatter plots show the effects of single nucleotide polymorphisms (SNPs) on IDPs against their effects on PD. The model-fit lines indicate the MR estimates of different MR methods.

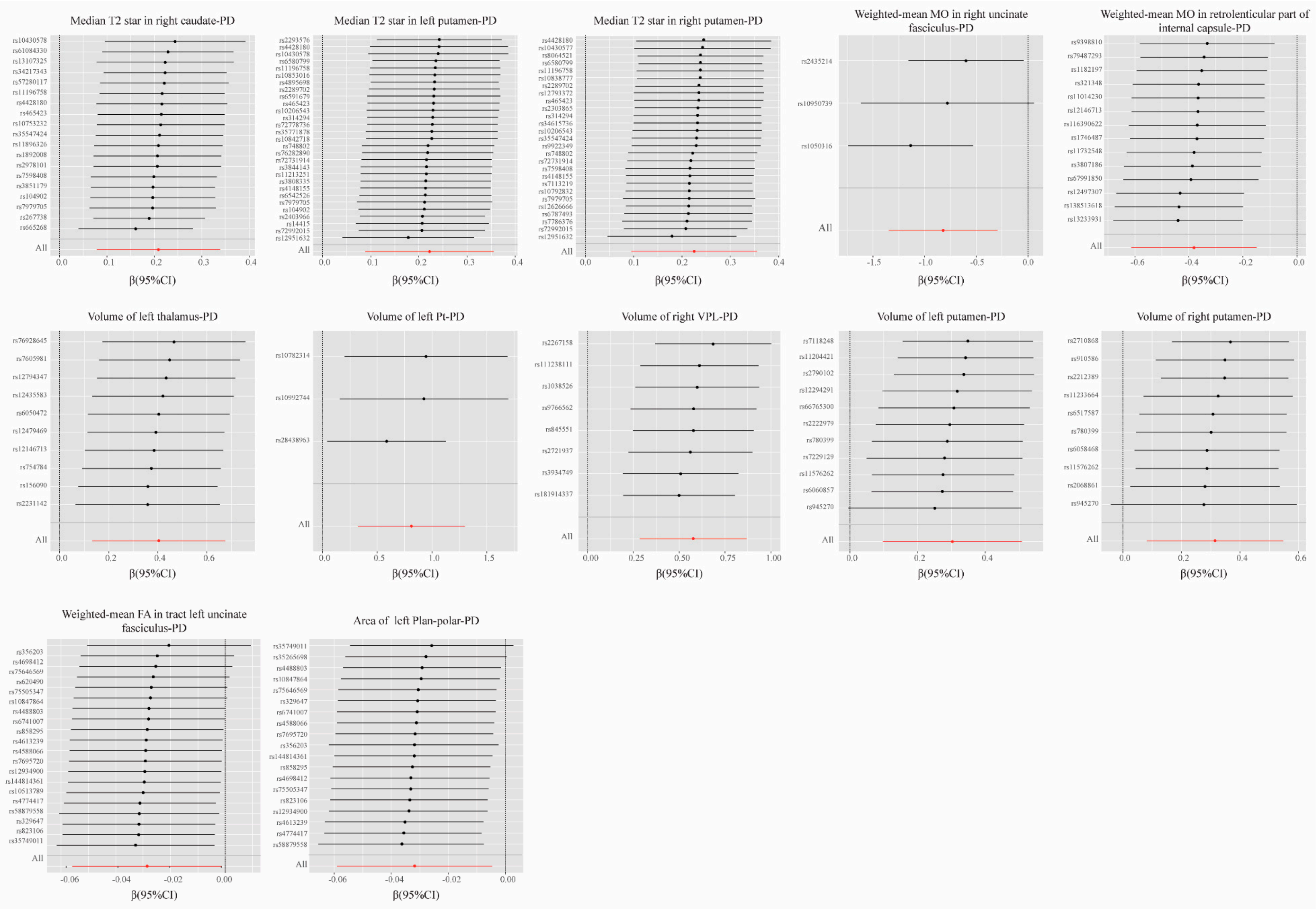

Figure S4. Leave-one-out plots of the forward Mendelian randomization study on the causal associations between IDPs and PD. Leave-one-out sensitivity analyses were used to identify potentially influential single nucleotide polymorphisms that exerted significant impacts on the associations. The horizontal line represents the 95% confidence intervals (CI) for the estimates.
