# Supplementary material for: Understanding Causal Relationships Between Imaging-Derived Phenotypes and Parkinson’s Disease: A Mendelian Randomization and Observational Study
Source: Biomedicines. 2025 Mar 18;13(3):747. doi: 10.3390/biomedicines13030747 (PMC11940266; doi:10.3390/biomedicines13030747)
Supplement: Supplementary file 1 [file biomedicines-13-00747-s001.zip › Supplementary Methods.pdf]

Supplementary Methods

Rationale for the Selection of Brain Imaging-Derived Phenotypes

To ensure biological relevance and statistical robustness, we selected imaging-derived phenotypes (IDPs) from the UK Biobank imaging cohort based on prior evidence of their association with Parkinson's disease (PD) and their potential to serve as plausible markers of neurodegeneration.

We incorporated IDPs from multiple imaging modalities, encompassing macrostructural, microstructural, and functional metrics, to provide a comprehensive assessment of PD-related brain alterations. Macrostructural measures included volumetric assessments at the global brain level and within key PD-associated regions, such as gray matter atrophy in cortical and subcortical areas, progressive cortical thinning, and reduced regional surface areas. Additionally, we specifically examined thalamic volumes, both bilaterally and within distinct subregions, given the functional heterogeneity of thalamic nuclei and their potential role in increasing PD risk.

For white matter integrity, we included microstructural measures such as fractional anisotropy, mean diffusivity, and intracellular volume fraction, as white matter degeneration has been implicated in PD pathophysiology. To ensure a comprehensive evaluation of white matter connectivity, we utilized data derived from Tract-Based Spatial Statistics and probabilistic tractography, minimizing the risk of missing relevant structural changes.

Furthermore, we incorporated susceptibility-weighted imaging-derived T2star values, which provide insights into brain iron deposition, a well-established hallmark of PD pathology. Given that excessive iron accumulation in deep brain structures is a key contributor to neurodegeneration in PD, these measures enhance our understanding of disease mechanisms.

To investigate functional connectivity alterations in PD, we included resting-state functional MRI (rs-fMRI) with Independent Component Analysis (ICA). Resting-state fMRI has been widely used to reveal disruptions in intrinsic brain network activity in PD, particularly in circuits governing motor control, cognition, and emotional regulation. As a data-driven approach, ICA effectively extracts independent functional components, enabling the identification of PD-related network dysfunctions without requiring predefined regions of interest.

Our selection was guided by previous neuroimaging studies demonstrating strong associations between these IDPs and PD pathogenesis and progression, including their relationships with motor and non-motor symptoms, such as cognitive impairment, sleep disturbances, and depression. Additionally, to enhance data reliability and comparability, we prioritized widely used measurement techniques and implemented rigorous quality control procedures to minimize bias and redundancy. This structured selection approach ensures that our study captures clinically meaningful neuroimaging markers while maintaining statistical rigor.

Selection of instrument variants and harmonisation of single nucleotide polymorphisms

In the two-sample MR investigation presented herein, we meticulously selected Instrumental variables (IVs) satisfying three core assumptions: (1) Strong Association with Exposure: The selected IVs demonstrate a strong association with the exposure. This exposure–IV linkage is statistically significant when the p value is less than 5×10^{-8}. (2) Independence from Potential Confounders: The IVs exhibit no correlation with potential confounders. Determination of each SNP's independence was achieved through a clumping algorithm conducted in PLINK that used the 1000 Genomes

Project as a reference point for Linkage Disequilibrium trimming. Consequently, the included SNPs maintained values of $R^2 < 0.001$ and LD > 10000 kb, signifying independence. Additionally, R^2 can be defined mathematically as MAF * (1 − MAF) * β^2*2. Cross-referencing selections with Phenoscanner V2 aided in assessing whether SNP-associated phenotypes might represent confounders for PD. (3) Pathway Restricted Influence on Outcome: IVs impact the outcome strictly via their influence on the intended exposure variable. Our final selection mandated that p2 values, indicative of the correlation between IVs and outcomes, exceeded 5×10^{-5}, thereby ensuring that IVs influence outcomes only through exposure.

For each stipulation, measures were carefully applied to assure that our instrumental variable selection contributes to the robustness and validity of this two-sample MR investigation.

For the quality control of instrumental variables (IVs), we further computed the F-statistic for each IV using the formula $F = R^2 * (\text{sample size} - 2) / (1 - R^2)$. We excluded those IVs with an F-statistic less than 10, suggesting that their associations with the exposure were insufficiently strong. Subsequently, we harmonized the data, removing palindromic and ambiguous SNPs. The IVs retained for our MR analyses are enumerated in Supplementary Tables S3 and S4.

Brain magnetic resonance imaging acquisition and processing

The acquisition of structural and functional images was conducted employing 3T Siemens scanners, equipped with a 12-channel head coil, at the Department of Radiology of Ruijin Hospital (Siemens Healthineers, Erlangen, Germany) . Before the commencement of scanning procedures, necessary safety protocols were diligently implemented to alleviate any potential hazards. A 3D magnetization prepared rapid acquisition gradient-echo (MPRAGE) sequence was used to acquire T1-weighted images with the following settings: slices = 192, field of view = 250 mm², thickness = 1.0 mm, flip angle = 9°, voxel size = 0.5 × 0.5 × 1 mm³, echo time = 2.44 ms, repetition time = 1900 ms, and inversion time = 900 ms. Structural connectivity was acquired using DWI scanning, with slices = 42, field of view = 220 mm², thickness = 3.0 mm, voxel size = 1.7 × 1.7 × 3.0 mm³, echo time = 94 ms, repetition time = 6000 ms, b-values = 0, and 1000 s/mm², and diffusion gradient directions = 30. Imagings were processed according to the UKB pipeline (https://git.fmrib.ox.ac.uk/falmagro/UK_biobank_pipeline_v_1, accessed on 4 October 2023).

Analyzing differences among groups in the observational study

A linear mixed-effects model was used to analyze group differences in T2star values or volume of specific brain regions, accounting for repeated measures across different brain regions within each subject. The independent variable was Group (three levels: HC, iRBD, and PD), and the dependent variable was T2star value (or volume). Brain Region (six regions: both sides of the caudate, putamen, and pallidum in the first analysis of T2star; three regions: the left thalamus, right VPL, and left Pt in the second analysis of volume) was treated as a nested factor within Group, considering potential structural differences. Age and Sex were included as covariates to control for possible confounding effects. The initial model was defined as follows:

$$T2^*/Volume_{ij} = \beta_0 + \beta_1 \text{group}_{ij} + \beta_2 \text{region}_{ij} + \beta_3 (\text{group} * \text{region})_{ij} + \beta_4 \text{sex}_{ij} + \beta_5 \text{age}_{ij} + u_i + \epsilon_{ij}$$

where $T2^*/Volume_{ij}$ represents the dependent variable, T2star value or brain region volume for a subject, in region j within group i. The parameter β_0 is the intercept, $\beta_1 \text{group}_{ij}$ represents

the fixed effect of group, $\beta_2\text{region}_{ij}$ corresponds to the fixed effect of region nested within group, $\beta_3(\text{group}*\text{region})_{ij}$ represents the interaction effect of group and region, u_i accounts for subject-specific random effects, and $\beta_4\text{sex}_{ij}$ and $\beta_5\text{age}_{ij}$ capture the effects of age and sex as covariates. The residual term ϵ_{ij} represents unexplained variability.

The initial full model included Group, Region, and the Group × Region interaction as fixed effects. Each subject was modeled with a random intercept to account for individual-level variability across repeated measurements. Type III F-tests were used to evaluate fixed effects. Since the Group × Region interaction as well as covariates age and sex were not statistically significant, they were removed, and a simplified model with Group and Region as main effects was used. The adjusted model was defined as follows:

$$\text{Value}_i = \beta_0 + \beta_1\text{group}_i + u_i + \epsilon_i$$

where Value_i represents the dependent variable, T2* value of brain region volume for a subject within group i .The parameter β_0 is the intercept, $\beta_1\text{group}_i$ represents the fixed effect of group, u_i accounts for subject-specific random effects. The residual term ϵ_i represents unexplained variability.

Regarding Thickness of left planum polare, independent analysis was conducted to assess whether these values differed across groups while controlling for age and sex. A linear mixed-effects model was used:

$$\text{Value}_i = \beta_0 + \beta_1\text{group}_i + u_i + \epsilon_i$$

where Value_i represents the dependent variable, T2star value of brain region volume for a subject within group i .The parameter β_0 is the intercept, $\beta_1\text{group}_i$ represents the fixed effect of group, u_i accounts for subject-specific random effects. The residual term ϵ_i represents unexplained variability.

After fitting the final model, we carried out post-hoc comparisons to explore significant effects in detail. Specifically, for the factor Group (with three levels) we were interested in pairwise differences between groups. A Tukey-Kramer adjustment was applied to these multiple comparisons to control the Type I error rate across all group pairs. All hypothesis tests were two-tailed, and a significance level of $\alpha = 0.05$ was used throughout.
